# FK506 and *Lactobacillus acidophilus* ameliorate acute graft-*versus*-host disease by modulating the T helper 17/regulatory T-cell balance

**DOI:** 10.1186/s12967-022-03303-z

**Published:** 2022-02-25

**Authors:** Jin-Ah Beak, Min-Jung Park, Se-Young Kim, JooYeon Jhun, Jin Seok Woo, Jeong Won Choi, Hyun Sik Na, Soon Kyu Lee, Jong Young Choi, Mi-La Cho

**Affiliations:** 1 The Rheumatism Research Center, Catholic Research Institute of Medical Science, College of Medicine, The Catholic University of Korea, 222, Banpo-daero, Seocho-gu, Seoul, 06591 Republic of Korea; 2 Department of Biomedicine & Health Sciences, College of Medicine, The Catholic University of Korea, 222, Banpo-daero, Seocho-gu, Seoul, 06591 Republic of Korea; 3 Department of Medical Lifescience, College of Medicine, The Catholic University of Korea, 222, Banpo-daero, Seocho-gu, Seoul, 06591 Republic of Korea; 4 Division of Hepatology, Department of Internal Medicine, Seoul St. Mary’s Hospital, College of Medicine, The Catholic University of Korea, 222, Banpo-daero, Seocho-gu, Seoul, 06591 Republic of Korea

**Keywords:** *L. acidophilus*, FK506, Th17 cell, Regulatory T cell, Allogeneic response, GvHD, Liver transplantation

## Abstract

**Background:**

Graft-*versus*-host disease (GvHD) is a critical complication after allogeneic hematopoietic stem cell transplantation (HSCT). The immunosuppressants given to patients undergoing allogeneic HSCT disturb the microbiome and the host immune system, potentially leading to dysbiosis and inflammation, and may affect immune function and bone marrow transplantation. The intestinal microbiome is a target for the development of novel therapies for GvHD. *Lactobacillus* species are widely used supplements to induce production of antimicrobial and anti-inflammatory factors.

**Methods:**

We determined the effect of the combination of *Lactobacillus acidophilus* and FK506 on GvHD following major histocompatibility complex-mismatched bone marrow transplantation.

**Results:**

The combination treatment suppressed IFN-γ and IL-17-producing T cell differentiation, but increased Foxp3^+^Treg differentiation and IL-10 production. Also, the combination treatment and combination treated-induced Treg cells modulated the proliferation of murine alloreactive T cells in vitro. Additionally, the combination treatment upregulated Treg-related genes—*Nt5e*, *Foxp3*, *Ikzf2*, *Nrp1* and *Itgb8*—in murine CD4^+^-T cells. The combination treatment also alleviated GvHD clinically and histopathologically by controlling the effector T cell and Treg balance in vivo. Moreover, the combination treatment decreased Th17 differentiation significantly and significantly upregulated Foxp3 and IL-10 expression in peripheral blood mononuclear cells from healthy controls and liver transplantation (LT) patients.

**Conclusions:**

Therefore, the combination of *L*. *acidophilus* and FK506 is effective and safe for patients undergoing allogeneic hematopoietic stem cell transplantation.

## Background

Allogeneic hematopoietic stem cell transplantation (allo-HSCT) is a remedial treatment modality for most hematologic malignancies. Its main complication, graft-*versus*-host disease (GvHD) affects the skin, liver, and gastrointestinal tract. GvHD is mediated by donor T cells in the graft and leads to a high rate of transplantation-related mortality [1, 2].

Tacrolimus (also known as FK506), a calcineurin inhibitor, is an anti-T-cell agent that is among the most widely used immunosuppressants [3]. FK506 inhibits calcineurin phosphatase and calcium-dependent events by binding to FK506 binding protein 12 (FKBP12), thereby preventing the dephosphorylation of nuclear factor of activated T-cells (NFAT) family members responsible for transcription of the T-cell–activating cytokines interleukin-2 and -4 [4–7]. However, the use of FK506 is associated with nephrotoxicity and can lead to dysbiosis [8–10].

The altered intestinal microbiota of patients with GvHD is correlated with GvHD severity and pathogenesis [11–13]. Intestinal bacteria of the order *Clostridiales* are important mediators of intestinal homeostasis [14–16] and the loss of intestinal bacteria of the genus *Blautia*, a member of the order *Clostridiales*, is associated with an increased mortality rate from GvHD [17]. Therefore, controlling the intestinal microbiota composition may be useful for preventing and treating GvHD [18].

Supplementation of probiotics may benefit gut-related immunity [19]. In mice, *Lactobacillus rhamnosus* reduced histologic inflammation and decreased the mortality rate post-transplantation [20]. *Lactobacillus* species exert an anti-inflammatory effect in vitro, and elimination of *Lactobacillus* species prior to allo-HSCT correlates with increased GvHD severity [21, 22].

We hypothesized that treatment with the combination of *L*. *acidophilus* and FK506 would ameliorate inflammation and GvHD. We evaluated the effect of *L*. *acidophilus* plus FK506 on GvHD in vitro and in vivo*.*

## Methods

### Mice

C57BL/6 (B6, H-2 kb) and BALB/c (B/c, H-2k^d^) mice at 8–10 weeks of age were purchased from OrientBio (Sungnam, Korea). The Institutional Animal Care and Use Committee (IACUC) and Department of Laboratory Animal of the Catholic University of Korea (Songeui Campus) accredited the Korea Excellence Animal laboratory Facility from Korea Food and Drug Administration in 2017 and acquired full accreditation by AAALAC International in 2018. Animal procedures were performed in accordance with the Laboratory Animals Welfare Act, the Guide for the Care and Use of Laboratory Animals, and the Guidelines and Policies for Rodent Experiments of the IACUC of the School of Medicine of The Catholic University of Korea (Approval number: CUMC-2020-0177-01).

### Patients

For this study, Liver transplantation (LT) patients were prospectively enrolled from a single LT clinic at Seoul St. Mary’s Hospital. The inclusion criteria were: more than 18 years old, liver transplanted more than three years ago; with 0–1 HLA-A, -B, and –DR mild mismatched; a high level of liver function with no history of rejection. Eight patients are being treated with CNIs including cyclosporine and tacrolimus. This study was approved by the Institutional Review Board of the Catholic University of Korea (KC19OESI0617).

### Murine splenocyte and human peripheral blood mononuclear cell culture and stimulation in vitro

Spleens from B6 mice were homogenized and red blood cells were lysed with ACK lysis buffer (0.15 M NH_4_Cl, 10 mM KHCO_3_, and 0.1 mM ethylenediaminetetraacetic acid; pH 7.2–7.4). Splenocytes were filtered through a cell strainer, centrifuged at 1300 rpm at 4 °C for 5 min, and resuspended in Roswell Park Memorial Institute (RPMI) 1640 medium supplemented with 5% [v/v] heat-inactivated fetal bovine serum (FBS). Human peripheral blood mononuclear cells (PBMCs) were isolated from human blood samples. Human blood samples by adding phosphate-buffered saline (PBS) was placed onto a layer of 10 mL Ficoll Plaque Plus (GE Healthcare Life Sciences, Marlborough, MA, USA) and centrifuged at 2000 rpm at 20 °C for 30 min. After centrifugation, PBMCs were washed and maintained in RPMI 1640 medium containing 10% FBS. A single suspension was prepared, and 1 × 10^6^ cells/well in 24-well flat bottom plates were cultured in the presence of plate-bound 0.5 μg/mL anti-CD3. Next, the cells were treated with *L*. *acidophilus* LA1 (100 ug/mL) and FK506 (0.3 nM for splenocytes; 1.0 nM for PB.

MCs) for 3 days. *L. acidophilus* was made inactive by heating at 80 °C, 30 min.

### Murine T-cell isolation and conditioning of alloreactive T-cell responses

To purify mouse splenic CD4^+^-T cells and antigen-presenting cells (APCs), splenocytes were incubated with CD4-coated magnetic beads and isolated by magnetic-activated cell sorting (Miltenyi Biotec, Bergisch Gladbach, Germany). Splenic CD4^+^-T cells from B6 (or B/c) mice were used as stimulator cells in the context of allorecognition. APCs from B/c (or B6) mice were used as the responder cells. Aliquots of 2 × 10^5^ CD4^+^-T cells (responders) were cultured with 2 × 10^5^ irradiated (5000 cGy) stimulators in 96-well plates containing 200 μL/well of complete medium, followed by treatment with 100 ug/mL *L*. *acidophilus* and/or 0.3 nM FK506 (for splenocytes) at 37 °C in a humidified 5% (v/v) CO_2_/air atmosphere for 4 days. The cells were pulsed with 1 μCi of tritiated thymidine (3[H]-TdR) (NEN Life Science Products, Boston, MA, USA) 18 h before harvesting using an automated harvester (PHD Cell Harvester; Cambridge Technology, Cambridge, MA, USA), and enumerated using a β-counter (Packard TopCount NXT). Data are expressed as mean cpm values of triplicate samples ± SEM.

### In vitro co-culture systems

To determine the blocking ability of alloreactive T-cell responses by *L. acidophilus* and/or FK506-induced Treg cells, we sorted the induced Treg cells and co-cultured them in alloreactive condition. Anti-CD3-stimulated CD4^+^-T cells were cultured with *L. acidophilus*, FK506 and combination of *L. acidophilus* and FK506. Three days later, cells of each condition were immunostained with anti-CD4 and anti-CD25 (eBioscience) and the induced Treg cells were sorted using a FACS Aria Fusion (BD Biosciences). Responder cells (2 × 10^5^) and irradiated APCs (2 × 10^5^) were co-cultured with each induced Treg cells (5 × 10^3^; each induced Treg to responder cells ratio is 1:40) in 96-well plates for 4 days, pulsed with 1 μCi tritiated thymidine at 18 h before the end of the experiment, and counted using an automated harvester, and enumerated using a β-counter (Packard TopCount NXT). Data are expressed as mean cpm values of triplicate samples ± SEM.

### Mouse bone marrow transplantation and scoring

To induce GvHD, splenocytes (5 × 10^6^) and bone marrow cells (5 × 10^6^) were isolated from donor (B6) mice and transplanted into recipient (B/c) mice by intravenous injection after 690 cGy irradiation. After induction of GvHD, recipient mice were orally administered *L*. *acidophilus* (1.6 × 10^10^ CFU/kg) and/or FK506 (5 mg/kg) daily. Control GvHD mice received vehicle (saline) in the same manner. The experiments were performed in at least triplicate, with five mice per group. Survival after bone marrow transplantation (BMT) was monitored daily and GvHD severity was assessed twice weekly using a scoring system based on summing changes in weight loss, posture, activity, fur texture, and skin integrity. Clinical GvHD scores were assessed using a previously described scoring system [23].

### Flow cytometry

Murine splenic lymphocytes and PBMCs and human PBMCs were immunostained with fluorescently conjugated antibodies against CD4, CD8, CD25, IL-17, IFN-γ, Foxp3, and Fixable-dye (BD Biosciences, San Diego, CA, USA). Prior to intracellular staining, cells were stimulated for 4 h with phorbol myristate acetate (25 ng/mL) and ionomycin (250 ng/mL) (Sigma-Aldrich, St. Louis, MO, USA) and treated with Golgistop (BD Biosciences). Intracellular staining was performed using a Cytofix/Cytoperm Plus Fixation/Permeabilization Kit and Golgistop Kit (BD Biosciences). The transcription factor Foxp3 was stained using a Foxp3/Transcription Factor Staining Kit (eBioscience) following the manufacturer’s protocol. Flow cytometry was performed using a cytoFLEX Flow Cytometer (Beckman Coulter, Brea, CA, USA) and data were analyzed in FlowJo software (Tree Star).

### Enzyme-linked immunosorbent assays

The concentrations of IFN-γ, IL-17, and IL-10 in culture supernatants of splenocytes and human PBMCs stimulated with anti-CD3 were measured by sandwich enzyme-linked immunosorbent assay (ELISA) (Duoset; R&D Systems, Lille, France).

### RNA sequencing

CD4^+^-T cells were isolated from B6 mice and subjected to T-cell activation conditions with or without *L*. *acidophilus* and FK506. Next-generation RNA-sequencing (RNA-seq) was conducted to quantify mRNAs and performed using a Novaseq 6000 (Illumina). Expression data were preprocessed using the range migration algorithm followed by quantile normalization. The RNA-seq data have been submitted in NCBI’s Gene Expression Omnibus (GEO) and is accessible through GEO Series Accession Number GSE174214.

### Histological and immunohistochemical analyses

Mice were euthanized on day 42 after BMT and the organs were harvested, cryoembedded, and sectioned. Tissue specimens were fixed in 10% formalin buffer and embedded in paraffin. Sections (6 μm thick) were stained with hematoxylin and eosin and analyzed using a histologic scoring system. For immunohistochemical staining, sections were stained with primary antibodies against IFN-γ, IL-17, and IL-10 overnight at 4 °C, followed by a biotinylated secondary antibody and streptavidin-peroxidase mixture for 1 h (Thermo Fisher, San Diego, CA, USA). Color was developed by adding 3,3-diaminobenzidine (Dako, Carpinteria, CA, USA). A histopathology score was graded using the scoring system described by Kim et al. and Fukui et al. [23, 24].

### Real-time polymerase chain reaction

Total RNA was extracted using TRI reagent (Molecular Research Center, Inc., Cincinnati, OH, USA) according to the manufacturer’s instructions. Complementary DNA was synthesized using the First-Strand cDNA Synthesis Kit (Dyne Bio). A LightCycler 2.0 instrument (software version 4.0; Roche Diagnostics) was used for PCR amplification with The SensiFAST™ SYBR Hi-ROX Kit (Bioline) following the manufacturer’s instructions. The following primers were used to amplify human genes: Foxp3, 5′-CAC TGC CCC TAG TCA TGG T-3′ (sense) and 5′-GGA GGA GTG CCT GTA AGT GG-3′ (antisense); and IL-10, 5′-CCA AGC CTT GTC TGA GAT GA-3′ (sense) and 5′-TGA GGG TCT TCA GGT TCT CC-3′ (antisense). Transcript levels were normalized to that of β-actin.

### Statistical analysis

We performed one-way analysis of variance (ANOVA) with Bonferroni’s post hoc test for multiple comparisons at a significance level of *p* < 0.05 using Prism ver. 5.01 software (GraphPad Software Inc., San Diego, CA, USA). Data are presented as means ± SEM.

## Results

### Combination treatment modulates murine T-cell proliferation in vitro

**Fig. 1:**
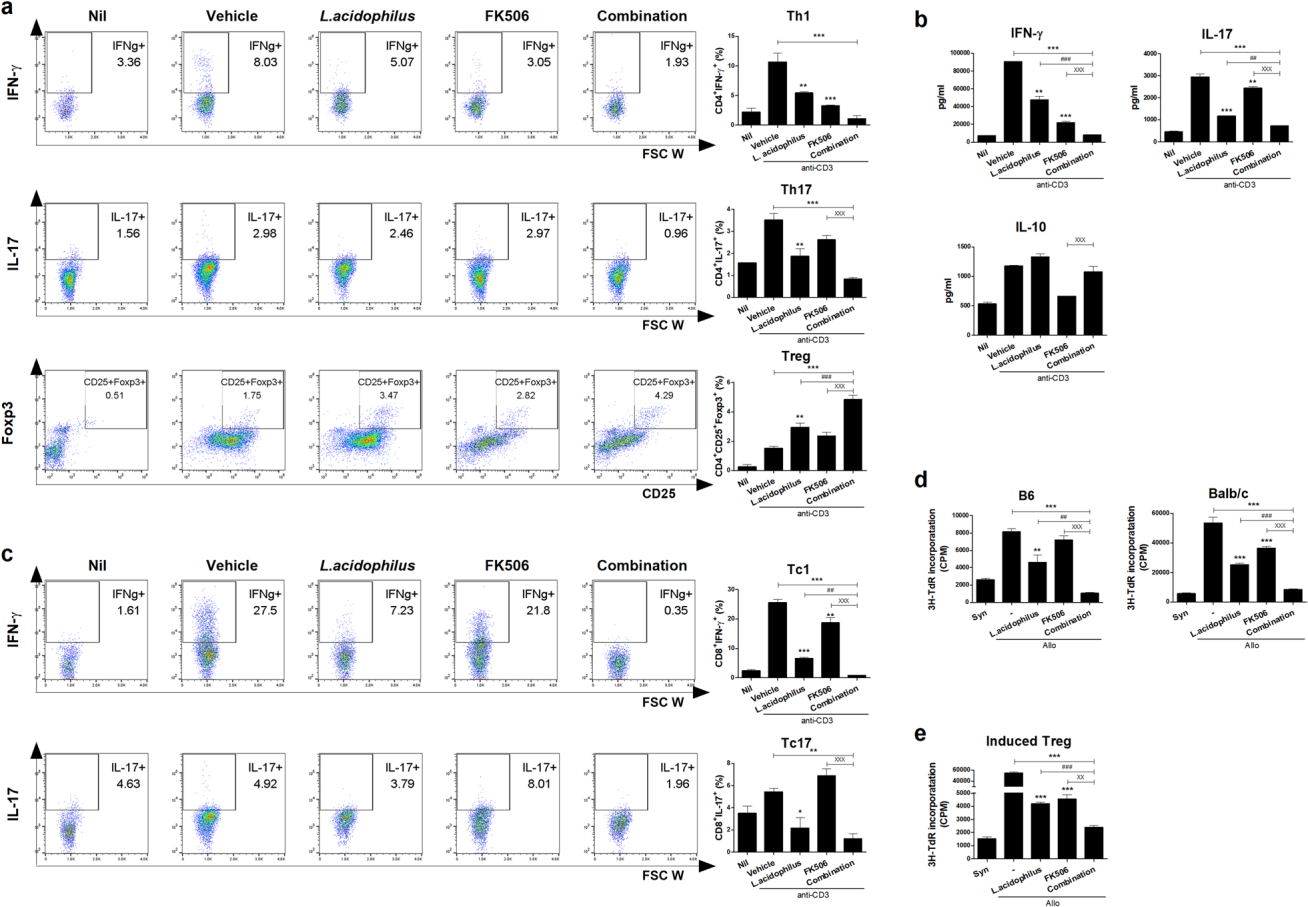
Combination treatment modulates mouse T cell subtype proliferation in vitro. **a** Proportions of Th1, Th17, and Treg cells after combination treatment. Splenocytes from normal B6 mice were stimulated with anti-CD3 in the presence of *L*. *acidophilus* and/or FK506 for 3 days and analyzed by flow cytometry. **b** IFN-γ, IL-17, and IL-10 concentrations in culture supernatants as determined by ELISA. **c** Proportions of Tc1 and Tc17 cells after combination treatment analyzed by flow cytometry. **d** In the mixed lymphocyte reaction assay, 2 × 10^5^ B/c (B6) splenic CD4^+^-T cells (responders) were incubated with 2 × 10^5^ irradiated B/c (B6) [syngeneic stimulators (Syn)] or B6 (B/c) [allogeneic stimulators (Allo)] splenic APCs for 4 days. T-cell proliferation was measured by 3[H]‑TdR incorporation. **e** In vitro co-culture systems, responder cells (2 × 10^5^) and irradiated APCs (2 × 10^5^) were co-cultured with sorted *L. acidophilus* and/or FK506-induced Treg (1 × 10^3^; each induced Treg to responder cells ratio was 1:40) for 4 days. T-cell proliferation was measured by 3[H]‑TdR incorporation. Data are means ± SEM of three independent experiments. (*each sample *versus* vehicle; ^#^combination *versus L*. *acidophilus*; ^X^combination *versus* FK506, *^,#,X^*p* < 0.05, **^,##,XX^*p* < 0.01, ***^,###,XXX^*p* < 0.005)

First, we investigated the effect of the combination treatment on murine IFN-γ-producing T cell, IL-17-producing T cell, and Treg cell differentiation in vitro. Splenocytes from normal B6 mice were cultured in the presence of an anti-CD3 antibody plus 100 ug/mL *L*. *acidophilus* and/or 0.3 nM FK506 for 72 h. The combination treatment significantly decreased Th1 and Th17 cell proliferation, and significantly increased the proportion of Treg cells, compared to vehicle and either single treatment (Fig. 1a). The concentrations of IFN-γ and IL-17 were decreased and that of IL-10 was significantly increased in combination-treated compared with vehicle-treated cells (Fig. 1b). Also, the combination treatment was significantly decreased CD8^+^ type 1 cytotoxic T cell (Tc1) and IL-17-producing CD8^+^ (Tc17) cell proliferation, and a single treatment with *L. acidophilus* was more effective than FK506 single on Tc 1 cells (Fig. 1c). To assess effects on the alloreactive T-cell response, we examined in vitro alloreactive T-cell proliferation in a mixed lymphocyte reaction. The combination treatment significantly suppressed alloreactive T-cell proliferation compared to allo and either single treatment (Fig. 1d). To confirm the effect of combination treatment, we sorted the Treg cells that induced by combination with *L. acidophilus* and FK506 and co-cultured in alloreactive condition. These induced Treg cells significantly suppressed alloreactive T-cell proliferation (Fig. 1e). Therefore, the combination treatment exerted an immunoregulatory effect on murine T cells in vitro.

### Effect of the combination treatment on gene expression in CD4^+^-T cells

**Fig. 2:**
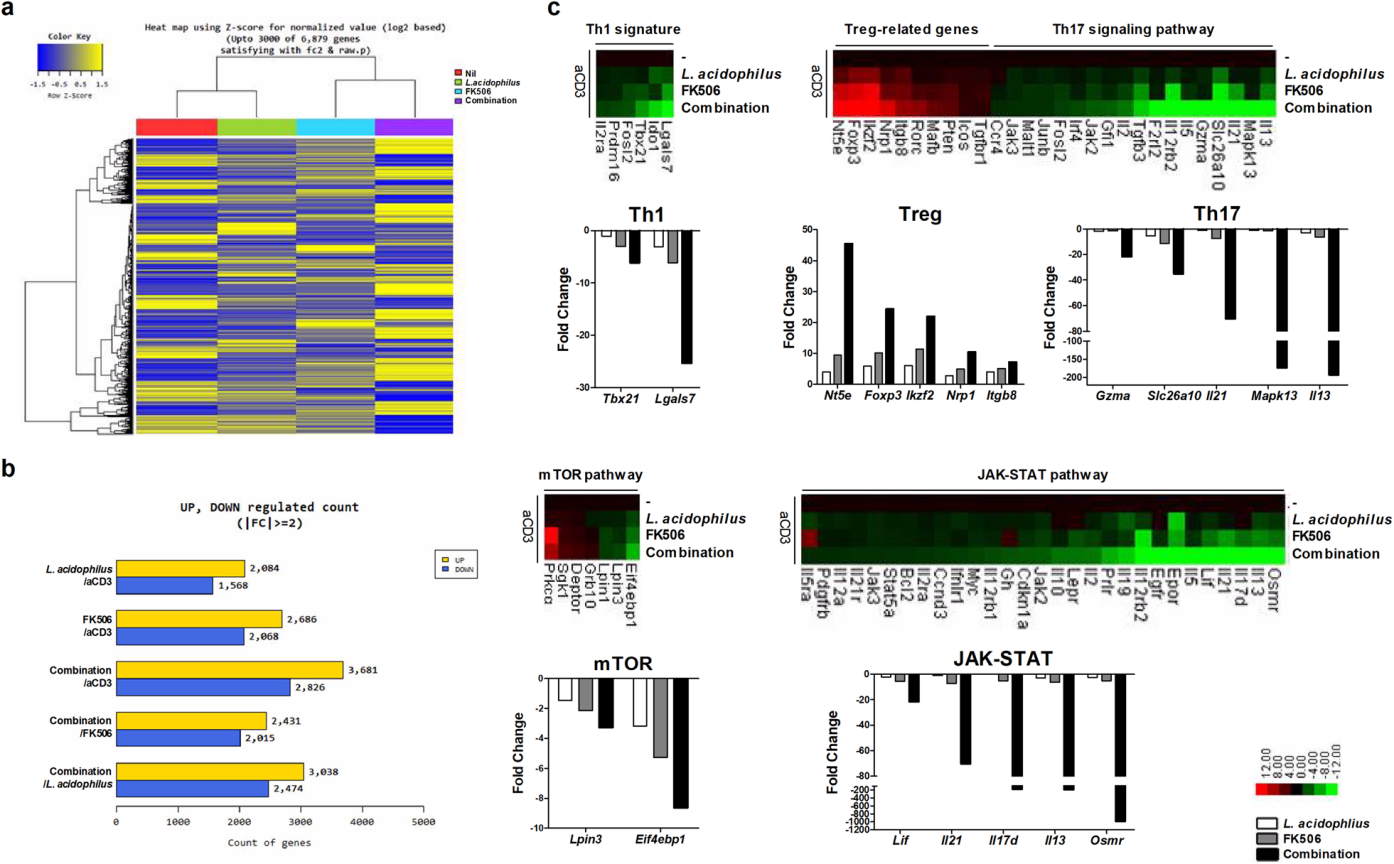
Gene expression profiling by RNA-seq among CD4^+^-T cells treated with *L*. *acidophilus*, FK506, and combination. **a** Hierarchical cluster heatmap of mouse anti-CD3 stimulated CD4^+^-T cells treated with *L*. *acidophilus* and/or FK506. **b** Significantly differentially expressed genes and **c** significant differences in Th1, Th17, and Treg populations and mTOR and JAK-STAT pathway activities *versus* only anti-CD3–treated CD4^+^-T cells

We next analyzed by RNA-seq the gene expression profiles of CD4^+^-T cells treated with *L*. *acidophilus* and/or FK506 in the presence of an anti-CD3 antibody (Fig. 2a). When identified by absent/present classification and using an at least two-fold difference in expression as the cut-off, 6507 genes were differentially expressed in anti-CD3–treated CD4^+^-T cells *versus* combination-treated CD4^+^-T cells (Fig. 2b). Among genes in the Th1 pathway, *Tbx21* and *Lgals7* were downregulated; among those in the Th17 pathway, *Il13*, *Mapk13*, *Il21*, *Slc26a10*, and *Gzma* were downregulated by the combination treatment compared with *L*. *acidophilus* or FK506 alone. Moreover, Treg-related genes such as *Nt5e*, *Foxp3*, *Ikzf2*, *Nrp1*, and *Itgb8* were upregulated in combination-treated CD4^+^-T cells. Regarding Th17 differentiation-related pathway genes linked to cellular growth and proliferation, *Eif4ebp1* and *Lpin3* (mTOR pathway) and *Osmr*, *Il13*, *Il17d*, *Il21*, and *Lif* (JAK-STAT pathway) were downregulated by the combination treatment (Fig. 2c). Taken together, these data suggest that combination-treated CD4^+^-T cells have an immunoregulatory function through the expression of Treg-related genes.

### Combination therapy reduced the severity of GvHD

**Fig. 3:**
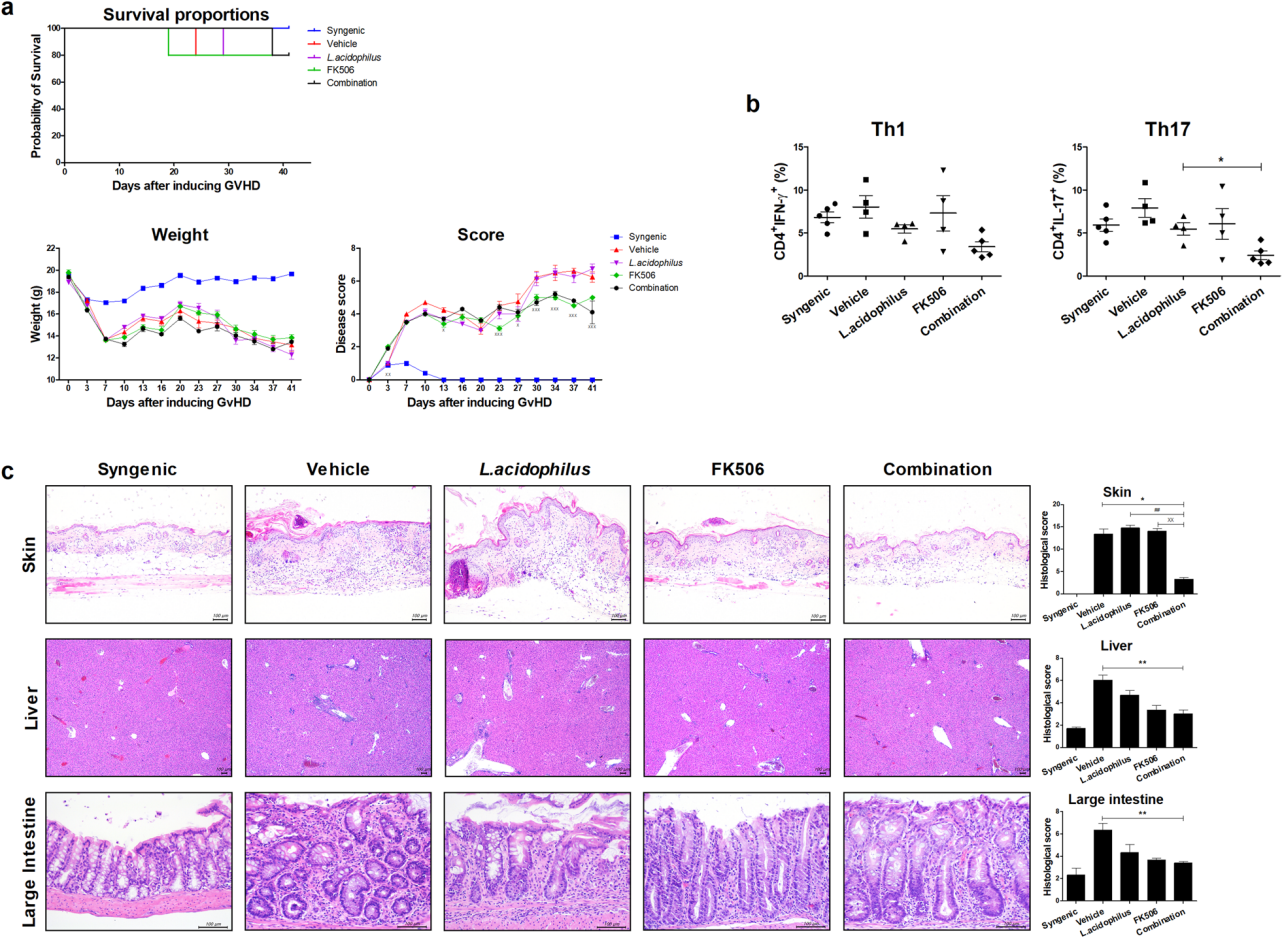
Combination treatment reduced the severity of GvHD. **a** Survival rate, weight loss, and clinical score after BMT in GvHD mice. Allogeneic‑transplanted animals showed a significantly increased GvHD score compared with syngeneic-transplanted animals. The clinical GvHD score was the sum of weight loss, posture, activity, fur, and skin on a scale of 0–2 (n = 5 per group and repeated three times). **b** Four weeks after BMT, IFN-γ and IL-17 expression in mouse CD4^+^-T cells as analyzed by flow cytometry. **c** Histopathology of the skin, liver, and large intestine after BMT (n = 5 per group) from one of two independent experiments. Sections were stained with hematoxylin and eosin (original magnification; liver × 40, skin and large intestine × 200; scale bar, 100 μm). Histopathology score of skin, liver, and large intestine tissues. Results are means ± SEM (*each sample *versus* vehicle; ^#^combination *versus L*. *acidophilus*; ^X^combination *versus* FK506, *^,#,X^*p* < 0.05, **^,##,XX^*p* < 0.01, ***^,###,XXX^*p* < 0.005)

To verify the effect of the combination treatment, bone marrow and splenocytes from a B6 donor were transplanted into a B/c recipient. The combination treatment improved the clinical symptoms of GvHD—weight loss, posture, activity, fur texture and skin integrity—compared with vehicle. The score of FK506 alone was similar to that of the combination, but the latter resulted in increased survival duration (Fig. 3a). Four weeks after BMT, we analyzed IFN-γ– and IL-17–producing CD4^+^-T cells in mouse PBMCs by flow cytometry. The populations of Th1 and Th17 cells were decreased by the combination treatment compared with vehicle or either single treatment (Fig. 3b). Next, we performed a histopathologic analysis of skin, liver, and large intestine tissues to determine the effect of the combination treatment on inflammatory cell infiltration. In the mouse model of GvHD, vehicle group has increased collagen density in skin, lymphocyte aggregates in portal tracts with destruction of bile ducts in liver tissue, and infiltration of lymphocyte and crypt loss of intestine, compared to syngenic [23, 24]. To determine the protective combination effects of *L. acidophilus* and/or FK506 on the development of GVHD, we evaluated tissue pathology in skin, liver and large intestine. As shown in Fig. 3c, moderate to severe GvHD was noted in those organs of vehicle group. Combination treatment group significantly improved pathologic severity scores in the skin, liver, and large intestine. Therefore, the combination treatment attenuated GvHD in vivo.

### Combination treatment modulates cytokine secretion

**Fig. 4:**
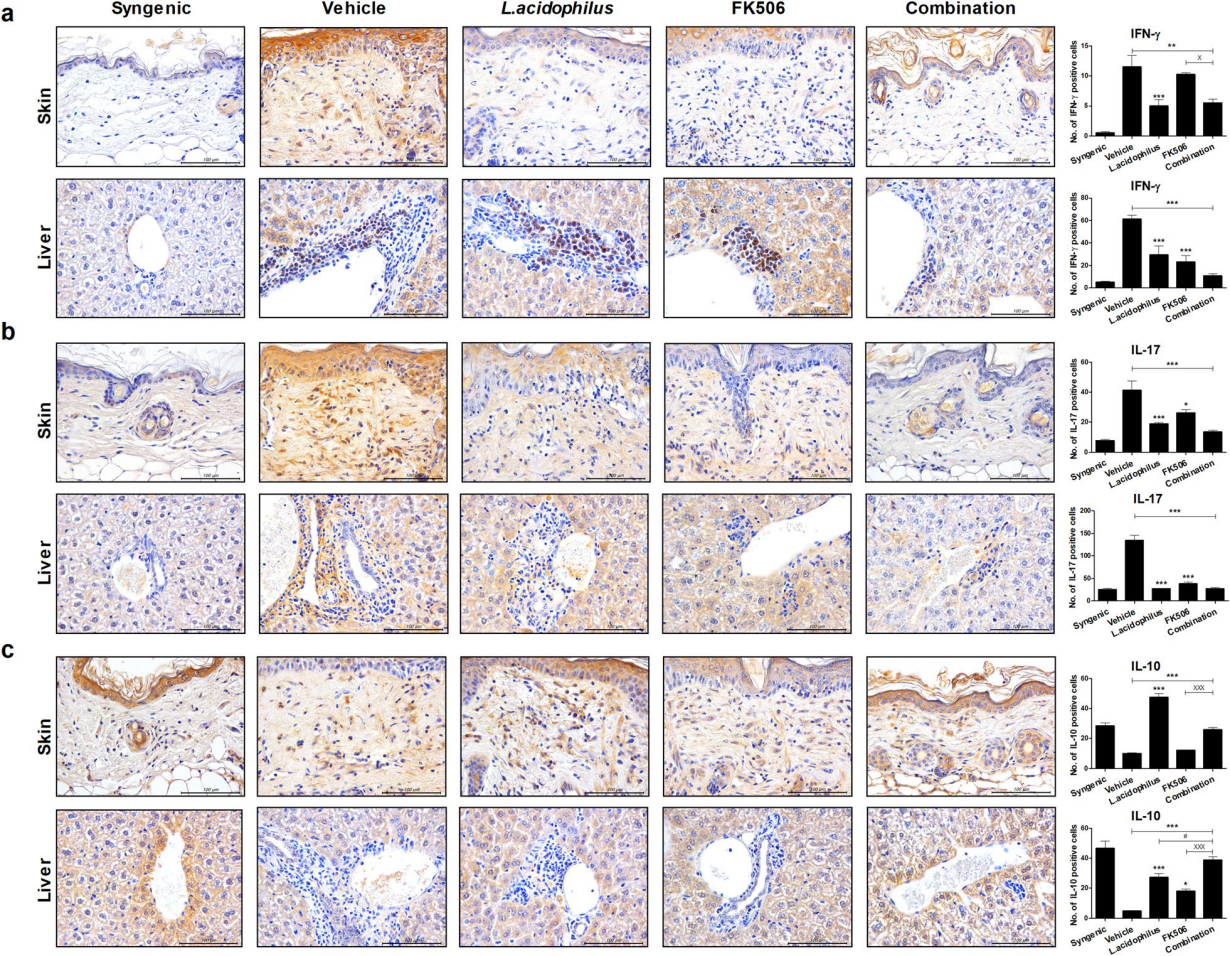
Combination treatment reduces and increases, respectively, the secretion of pro- and anti-inflammatory cytokines. Immunohistochemical images of tissues stained with **a** anti-IFN-γ, **b** anti-IL-17, and **c** anti-IL-10 antibodies (original magnification ×400, scale bars, 100 μm)

Next, we evaluated the expression levels of pro- and anti-inflammatory cytokines by immunohistochemistry in murine skin and liver tissues after BMT. The levels of proinflammatory cytokines, such as IFN-γ and IL-17, were significantly decreased by the combination treatment *versus* vehicle in skin and liver tissue. Also, the IFN-γ level in skin tissue was significantly decreased by the combination treatment compared with either single treatment (Fig. 4a, b). The concentration of IL-10, an anti-inflammatory cytokine, was significantly increased by the combination treatment compared with vehicle and either single treatment (Fig. 4c). Therefore, the combination of *L*. *acidophilus* and FK506 after BMT regulates T-cell infiltration of target tissues.

### Effect of the combination treatment on human PBMCs in vitro

**Fig. 5:**
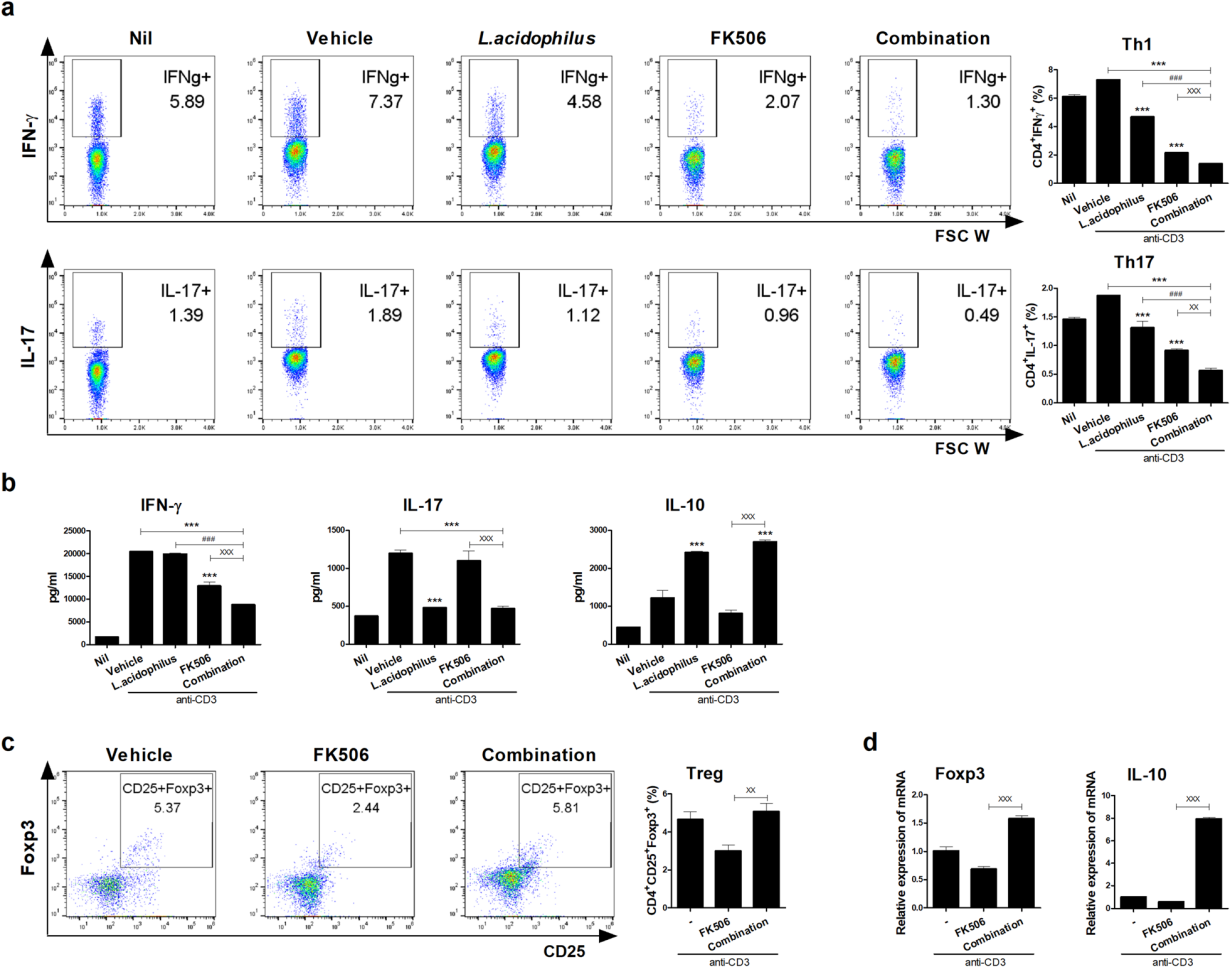
Effect of the combination treatment on healthy PBMCs in vitro. **a** Proportions of Th1 and Th17 cells. Human PBMCs from healthy donors were stimulated with anti-CD3 in the presence of *L*. *acidophilus* and/or FK506 for 3 days and analyzed by flow cytometry. **b** IFN-γ, IL-17, and IL-10 concentrations in culture supernatants as determined by ELISA. **c** After stimulation of healthy PBMCs with anti-CD3 for 3 days, proportion of Treg cells were determined by flow cytometry and **d** Foxp3 and IL-10 mRNA levels were determined by real-time PCR (*each sample *versus* vehicle; ^#^combination *versus L*. *acidophilus*; ^X^combination *versus* FK506, *^,#,X^*p* < 0.05, **^,##,XX^*p* < 0.01, ***^,###,XXX^*p* < 0.005)

**Fig. 6:**
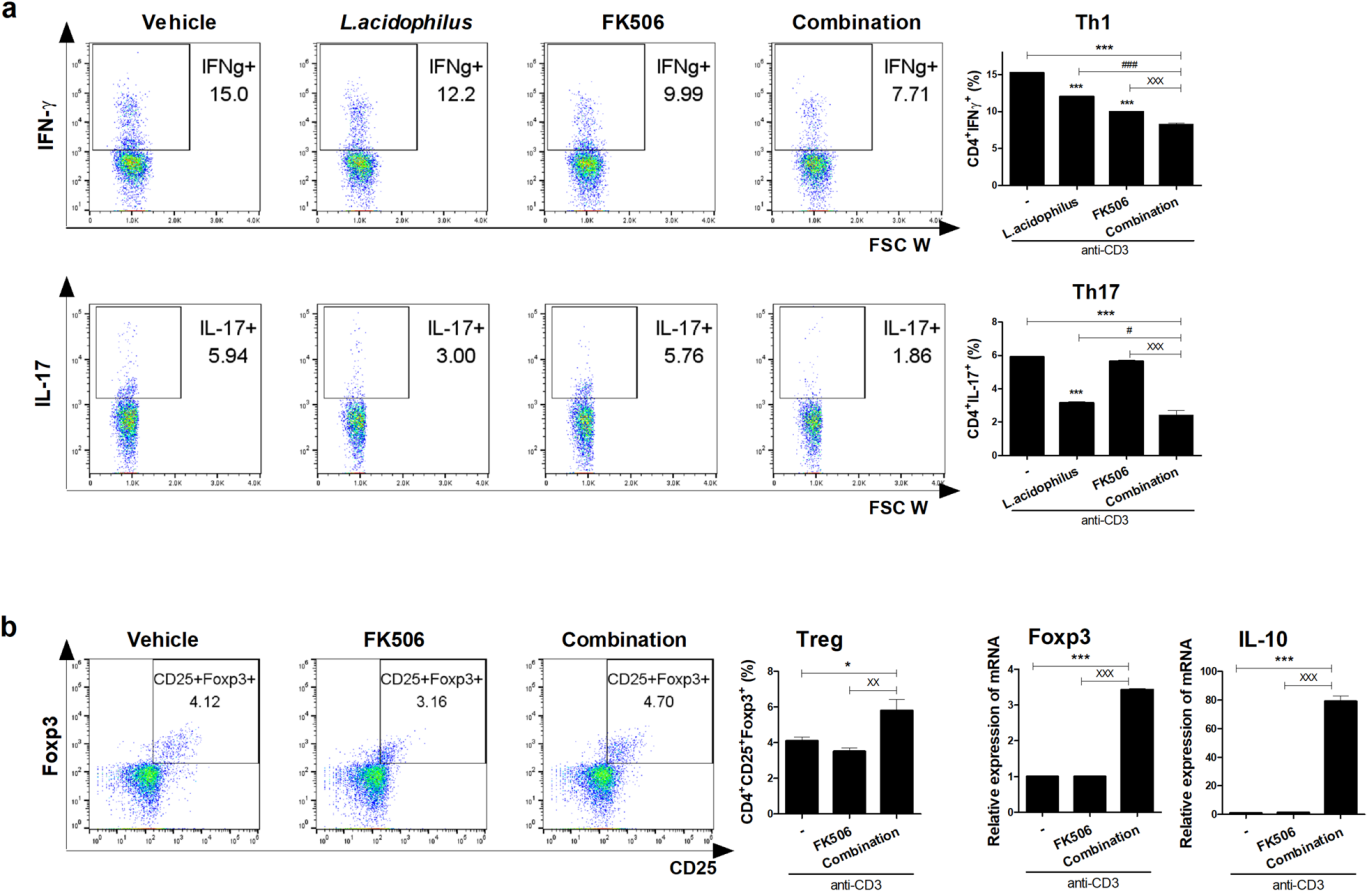
Effect of the combination treatment on LT-patient PBMCs in vitro. **a** Proportions Th1 and Th17 cells among PBMCs treated with *L*. *acidophilus* and/or FK506. Patient PBMCs were stimulated with anti-CD3 in the presence of *L*. *acidophilus* and/or FK506 for 3 days and analyzed by flow cytometry. **b** Proportion Treg cells were analysis by flow cytometry, and Foxp3 and IL-10 mRNA levels were determined by real-time PCR after stimulation of LT-patient PBMCs with anti-CD3 for 3 days. Data are means ± SEM of three independent experiments (*each sample *versus* vehicle; ^#^combination *versus L*. *acidophilus*; ^X^combination *versus* FK506, *^,#,X^*p* < 0.05, **^,##,XX^*p* < 0.01, ***^,###,XXX^*p* < 0.005)

To investigate the regulatory effect of the combination treatment on human Th1 and Th17 cell differentiation, PBMCs from healthy controls (n = 6) and LT patients (n = 8) were cultured under anti-CD3 conditions with *L*. *acidophilus* and/or FK506 for 72 h in vitro. Flow cytometry showed that the combination treatment significantly decreased Th1 and Th17 cell proliferation (Figs. 5a and 6a) and increased Treg cells compared with FK506 and vehicle (Figs. 5c, and 6b). The concentrations of IFN-γ and IL-17 were decreased and that of IL-10 was significantly increased by the combination treatment compared with FK506 or vehicle (Fig. 5b). Also, the transcript levels of Foxp3 and IL-10 were significantly increased by the combination treatment in PBMCs from healthy controls and LT patients (Figs. 5d, 6b). Therefore, the combination treatment suppressed human Th17-cell and promoted human Treg-cell proliferation in vitro.

## Discussion

Tacrolimus (FK506) is an immunosuppressant used to prevent allograft and treat autoimmune diseases [25–28]. However, immunosuppressive agents increase susceptibility to infections and alter the gut microbiota composition, the latter is associated with the severity and pathogenesis of GvHD [10–13, 29]. For this reason, maintaining the optimal concentration of tacrolimus is important for patient survival; however, determining the optimum concentration is hampered by interindividual variation in tacrolimus efficacy [30–32]. We hypothesized that *L*. *acidophilus* could ameliorate the side effects of FK506, and thus mitigate GvHD following MHC-mismatched bone marrow transplantation.

Probiotics supplementation is widely used in clinical practice, and promotes re-growth of beneficial species, ameliorating diseases such as IBD, colitis, RA, and osteoporosis [33–37]. IFN-γ-producing T cell such as Th1 and Tc1, IL-17-producing T cell such as Th17 and Tc17 are crucial mediators of GvHD [38–43] and GvHD is associated with a reversed proportion of Tregs [44, 45]. *Lactobacillus* species, probiotics widely used as health supplements, are commensal bacteria that reduce inflammation by stimulating lymphocytes via IL-10 [46, 47]. Furthermore, *L*. *acidophilus*, specifically the LA1 strain, is known to interact with intestinal cells to activate pathways that repair defective intestinal barriers and effectively treat intestinal inflammation [48]. In this study, the combination treatment significantly decreased the population of Th1, Tc1, Th17 and Tc17 cells (Figs. 1a, c, 5a, c, 6a and b) as well as the IFN-γ and IL-17 concentrations, but significantly increased the population of Treg cells and the IL-10 concentration, compared with FK506 alone (Figs. 1a, b, 5b, c and 6b) in murine and human. Moreover, the alloreactive T-cell response was decreased significantly by the combination treatment (Fig. 1d). However, the transcript levels of Foxp3 and IL-10 in human PBMCs were significantly increased by the combination treatment (Figs. 5d, 6b). Further study is required to confirm that combination treatment of *L. acidophilus* with FK506 can modulate Tc1 and Tc17 cell in vivo and human in vitro.

Dysbiosis of the gut microbiota influences immune tolerance and triggers autoimmune and inflammatory diseases by causing an imbalance of T cell subsets [49, 50]. *L*. *acidophilus* has immunomodulatory properties such as modulating Th17 and Treg cell populations. *L*. *acidophilus* induced production of Treg cells and IL-10, but suppressed that of IL-17, in splenocytes [35]. In addition, the immunoregulatory function of Tregs is used in the therapeutic strategies for autoimmune diseases such as SLE, RA and GvHD because mechanisms of Treg cells that have been modulation of cell proliferation [51–54]. In this study, combination treated-induced Treg suppressed the T cell proliferation (Fig. 1e) and the combination treatment reduced the effector T-cell population by modulating Th17, mTOR, and JAK-STAT signaling, and upregulating Treg-related genes, compared to *L*. *acidophilus* and FK506 alone (Fig. 2). In particular, Treg-related genes, *Nt5e*, *Foxp3*, *Ikzf2*, *Nrp1* and *Itgb8*, are up-regulated by combination treatment of *L. acidophilus* and FK506. *Nt5e*, also known as CD73, is known to have immunosuppressive potential through restricting inflammatory immune responses [55, 56]. *Foxp3* is essential for the maintenance of Treg and *Nrp1* is required to maintain Treg stability and function [57–59]. The deficiency of *Ikzf2* (as known as Helios) and *Itgb8* cannot control the expansion of pathogenic T cells during active inflammation [60, 61]. It could be suggested that the combination showed a greater effect than single treatment of FK506 because of the Treg-related genes. Therefore, *L*. *acidophilus* improved the immunological imbalance caused by FK506 by decreasing and increasing the Th17 and Treg cell populations in vitro.

*L*. *acidophilus* reduced the serum IL-6 and IL-17 levels and downregulated IL-17A expression, but upregulated CD25, Foxp3, and TGF-β expression in a murine model of allergy [62]. Also, *L*. *acidophilus* leads to the Treg-Th17 cell balance by inhibiting Th17 cells and promoting Treg cells in ovariectomized mice [63]. We used *L*. *acidophilus* because of its immunoregulatory activity, as confirmed in this study by its in vivo and in vitro immunoregulatory effects on Th17 and Treg cells in a GvHD model. Indeed, the combination treatment improved the clinical scores of mice with GvHD. Moreover, the combination therapy reduces the Th1 and Th17 cell populations compared with vehicle after 4 weeks in vivo (Fig. 3b). In GvHD target tissues, such as skin and liver, the levels of proinflammatory cytokines were significantly decreased and that of an anti-inflammatory cytokine was significantly increased by the combination treatment (Fig. 4). Therefore, the combination treatment suppressed inflammation and pathogenic cell activity.

Taken together, the results show that treatment with *L*. *acidophilus* or FK506 exerted similar effects, which were of lesser magnitude than those of the combination treatment. In other words, *L. acidophilus* served as a supplement to low-dose FK506 in a GvHD model and showed synergistic effects in this study.

## Conclusions

In conclusion, improvement of immune imbalance by probiotics has therapeutic potential for GvHD. The addition of *L*. *acidophilus* to FK506 reduced inflammation and the severity of GvHD compared to FK506 alone in vivo and in vitro. We propose that the combination treatment of *L. acidophilus* with FK506 could prevent GvHD progression through effector T cell and Treg cell modulation, also *L*. *acidophilus* could reduce the side effects and maximize the therapeutic efficacy of FK506 in allo-HSCT patients.

## Data Availability

All datasets generated for this study are included in the article.
